# Regulation of endothelial homeostasis, vascular development and angiogenesis by the transcription factor ERG

**DOI:** 10.1016/j.vph.2016.05.003

**Published:** 2016-11

**Authors:** Aarti V. Shah, Graeme M. Birdsey, Anna M. Randi

**Affiliations:** Vascular Sciences, Imperial Centre for Translational and Experimental Medicine, National Heart and Lung Institute, Imperial College London, London, United Kingdom

**Keywords:** ETS transcription factors, Gene transcription, Angiogenesis, Vascular development, Endothelial homeostasis

## Abstract

Over the last few years, the ETS transcription factor ERG has emerged as a major regulator of endothelial function. Multiple studies have shown that ERG plays a crucial role in promoting angiogenesis and vascular stability during development and after birth. In the mature vasculature ERG also functions to maintain endothelial homeostasis, by transactivating genes involved in key endothelial functions, while repressing expression of pro-inflammatory genes. Its homeostatic role is lineage-specific, since ectopic expression of ERG in non-endothelial tissues such as prostate is detrimental and contributes to oncogenesis. This review summarises the main roles and pathways controlled by ERG in the vascular endothelium, its transcriptional targets and its functional partners and the emerging evidence on the pathways regulating ERG's activity and expression.

## Graphical abstract

ERG regulation of endothelial function and homeostasis.

**Figure:**
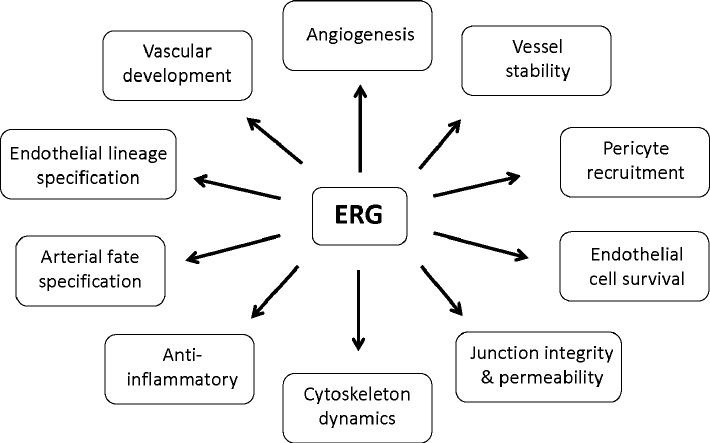

## 1 ETS family of transcription factors

The 28 mammalian ETS (for E-26 transformation specific) transcription factors share a highly conserved 85 amino acid DNA binding domain (ETS domain) that binds to a DNA core consensus motif 5′GGA(A/T)3′ [72]. Further specificity in binding is defined by the flanking bases; however the precise mechanisms that control ETS factor/DNA binding specificity are still unclear. This is a key question, given that multiple ETS factors can be expressed by the same cell at the same time. Another conserved domain shared by a number of ETS factors is the ~ 80 amino acid pointed domain (PNT), which has been shown to function as a site of interaction with kinases and transcriptional co-regulators, and is involved in dimerization with other ETS transcription factors [51], [86], [89]. The ability of ETS factors to act in concert with other transcription factors is exemplified by the presence of composite DNA binding sites, including FOXC/ETS and AP-1/ETS sites on target genes [18], [67].

ETS factors can act as transcriptional activators, repressors or both, depending on the target gene or post-translational modifications [55], [89]. Some ETS factors are expressed in a distinct temporal window of development, such as ETV-2 [108]; some, such as ERG, first appear during development and are maintained through adulthood (see below); others, such as ETS-1, are expressed in response to signals promoting inflammation or cell growth [61], [97], [114]. Some ETS factors, such as ELK-1, are ubiquitous [39] and mediate diverse cellular functions including cell growth, differentiation, proliferation, survival, cell–cell and cell–matrix interactions (reviewed in Ref. [72]). Others, such as ETS-1, ERG and FLI-1, have a restricted profile of expression and are important in the regulation of tissue-specific processes that include haematopoiesis, angiogenesis and vascular inflammation. Several ETS factors including ETS-1, ETS-2, PU-1 (SPI1), FLI-1, ERG and TEL (ETV6) can act as proto-oncogenes and have been implicated in the pathogenesis of different types of cancer (reviewed in Ref. [88]).

## 2 ETS factors in the endothelium

At least 19 ETS factors have been shown to be expressed in human endothelial cells (EC) at some point during development (reviewed in Ref. [79]). ETS factors are central to the transcriptional systems controlling EC gene expression, as all characterized endothelial promoters and enhancers contain ETS DNA-binding motifs, which can be bound by multiple ETS family members [19]. Several studies have shown that ETS factors are required to drive endothelial-specific gene expression. Functional ETS binding motifs have been identified within the promoters of endothelial-restricted genes, including vascular endothelial growth factor receptor (VEGFR)-1, VEGFR-2, TIE1, TIE2, endothelial nitric oxide synthase (eNOS) and VE-cadherin (also see Section 9.1). Many ETS factors are expressed in the vasculature of several organisms during development; both gain and loss-of-function studies in mice and zebrafish have shown a key role for ETS proteins during vascular development ([98], [109], [112], [124]; reviewed in Ref. [79]).

## 3 The ETS related gene ERG: genomic structure and isoforms

The ETS related gene (ERG) gene maps to the reverse strand of chromosome 21 (21q.22.2) [73], [80] and spans 282 kb with up to 12 potential exons. The human ERG gene has at least 3 recognized proximal promoters [100], [123]. Additionally, a region 85 kb downstream of the transcription start site has been identified as an ERG enhancer, which is active during normal haematopoiesis and in T-cell acute lymphoblastic leukaemia cells. ERG has been shown to positively regulate its own expression via the + 85 enhancer in these cells [100].

**Fig. 1:**
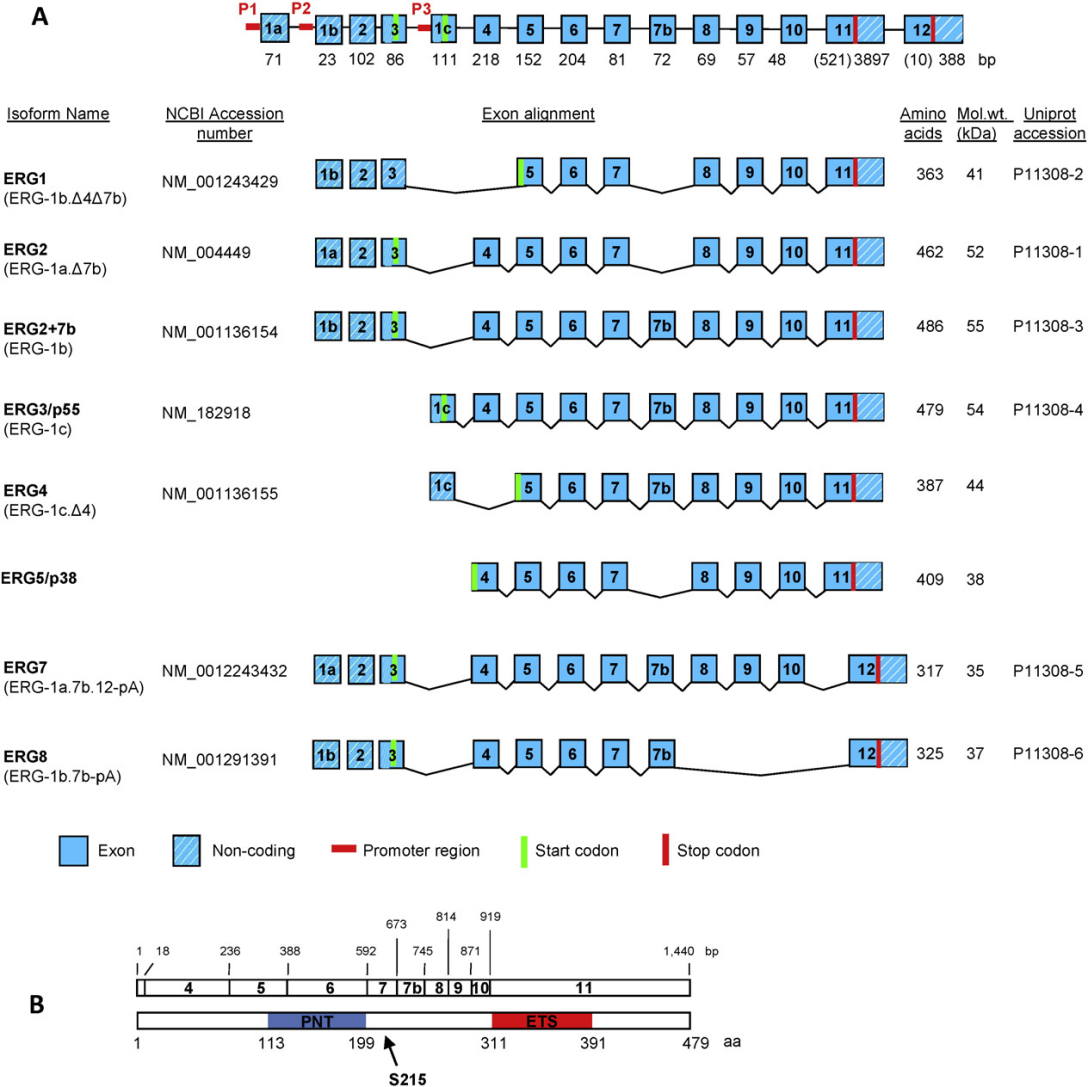
Structure of the human ERG gene and isoforms. (A) The major ERG exons are shown with their size in base pairs (bp) below each exon; numbers in parentheses indicate nucleotides within the open reading frame of the alternatively spliced exons 11 and 12. The three alternative promoters (P1, P2, P3) are indicated in red. Eight reported ERG isoforms are listed below along with their respective NCBI accession numbers (if available). The name for each isoform follows the commonly used nomenclature; in parentheses are the names proposed by Zammarchi et al. [123]. The predicted number of amino acids, predicted size in KDa, and Uniprot (Universal Protein Resource) accession numbers are shown to the right of the exon alignment. (B) The ERG3/p55 exon structure and nucleotide length (in base pairs) is aligned with the predicted protein sequence showing the amino acid position of the main protein domains. PNT (pointed domain), ETS (ETS DNA-binding domain). The phosphorylated serine residue at position 215 is indicated by an arrow. (Modified from Refs. [110], [123].)

A study carried out by Zammarchi et al. identified over 30 ERG isoform variants, leading to the potential production of at least 15 polypeptides, the longest of which encodes a protein of 486 amino acids with a molecular mass of 54.6 kDa [123]. Expression of the ERG isoforms is dependent on alternative exon splicing and on the use of alternative polyadenylation sites and translation initiation codons (Fig. 1A). Of the alternative ERG transcripts previously identified, ERG1, ERG2, ERG3 (p55), ERG4 (p49), and ERG5 (p38) encode for functional proteins that bind DNA [25], [77], [82]. ERG7 and ERG8 are predicted to form functional proteins as they have open reading frames; however both variants lack the C-terminal ETS DNA-binding domain [73]. Interestingly, a recent study identified a conserved nuclear localization sequence in the ERG ETS domain and showed that ERG8, which lacks the ETS domain, was unable to bind DNA and was mainly localized to the cytoplasm [38]. Although lacking transcriptional activity itself, ERG8 was shown to interact with other ERG isoforms to inhibit their transcriptional activity [38], [81]. Furthermore, knockdown of ERG8 in EC results in upregulation of endogenous ERG transcriptional activity, suggesting that ERG8 functions as an inhibitor of ERG's active isoforms [38]. Reverse transcriptase-PCR analysis using isoform-specific primers indicates that ERG3 and ERG5 are constitutively expressed in quiescent EC [36], with ERG1 and ERG8 expressed at much lower levels [38].

## 4 ERG DNA binding activity and functional domains

Analysis of deletion mutants has led to the characterization of ERG protein domains mediating DNA binding and transcriptional activation [92]. The ETS domain is located in the C-terminus of ERG (Fig. 1B), and as with other ETS factor family members, is essential for DNA binding. Multiple studies have investigated the ERG DNA binding consensus sequences flanking the core (GGAA/T) DNA consensus motif. Early studies using electrophoretic mobility shift assays (EMSA) identified specific ERG consensus sequences as (C/G)(C/a)GGAA(G/a)T [68] or (A/C)GGAAG [25]. Further genome-wide studies using chromatin immunoprecipitation coupled with high-throughput DNA sequencing (ChIP-seq) characterized the sequences AGGA(A/t)(G/A) [115] or (C/a/g)(A/C)GGAA(G/A/c) [113] as specific ERG consensus sequences. Interestingly, a recent study has shown that ERG DNA-binding is allosterically regulated by autoinhibitory regions both N- and C-terminally adjacent to the ETS domain [83].

ERG also possesses a second structured domain known as the pointed (PNT) domain (Fig. 1B), which is conserved in ten other ETS factors (ETS-1, ETS-2, FLI-1, GABPα, TEL (ETV6), TEL-2 (ETV7), ESE-1 (ELF3), ESE-2 (ELF5), ESE-3 (EHF) and PDEF (SPDEF)) [49]. The ERG PNT domain comprises four α-helices and a short α-helix [40]. Carrere et al. suggested a role for the PNT domain in mediating protein–protein interactions and homo/hetero-dimerization [16]. Deletion of the PNT domain has been shown to cause a 70% decrease in ERG2 transcriptional activity using a reporter assay in NIH3T3 cells [92]. ERG contains a C-terminal transcriptional activation (CTA) domain, which is also conserved in FLI-1; the transcriptional activation function of the CTA domain is repressed by a negative regulatory transcriptional activation (NRT) domain [92].

## 5 ERG binding partners and functional partners

ERG appears to functionally and/or physically interact with several transcription factors; a list of ERG known binding and functional partners is shown in Table 1. Carrere et al. reported that the ERG proteins can form homo and hetero-dimeric complexes in vitro [16]. The authors identified 2 domains involved in ERG dimerization: the ETS domain and a region within the amino-terminus of the protein containing the pointed domain. Furthermore, they showed that ERG can also form heterodimers with other ETS factors, including FLI-1, ETS-2 and PU-1 [16]. The ERG ETS domain also mediates the interaction with activator protein 1 (AP-1), a heterodimeric transcription factor composed of FOS and JUN proteins [15], [16], [103].

A yeast two-hybrid screen performed using the full-length *Xenopus* ERG protein as bait identified three binding partners: the homeobox transcription factors Xvent-2 and Xvent-2B and the small nuclear RNP C protein [22]. Yang et al. screened a yeast two-hybrid cDNA library constructed from mouse haematopoietic cells using the amino-terminal region of ERG as bait [117]. This study showed that ERG interacted with UBC9, a ubiquitin-conjugating enzyme and with ESET (ERG associated protein with a suppressor of variegation, enhancer of zest and trithorax domain), a histone H3-specific methyltransferase [117], which also interacts with the transcriptional co-repressors histone deacetylase 1 and 2 (HDAC1/2) and mSin3A/B [118]. Co-immunoprecipitation studies on tagged proteins expressed in COS-7 cells have shown that ERG is able to associate with the transcription factor KLF2 [63]. Transactivation studies in HeLa cells also suggest a functional interaction between ERG and the transcriptional co-activator p300 [44].

In prostate cancer cells, ERG was shown to physically interact with the enzymes poly(ADP-ribose) polymerase 1 (PARP1) and the catalytic subunit of DNA protein kinase (DNA-PKcs), which play a role in ERG-induced transcription in vCaP prostate cancer cell-line overexpressing the TMPRSS2:ERG fusion protein (see Section 10.3.2) [13]. ERG also forms a complex with the Ku70 and Ku80 subunits of the DNA repair enzyme Ku, in a DNA-dependent manner [13].

Like many transcription factors, ETS proteins control gene expression by combinatorial interaction between transcription factors and their binding motifs on DNA. Wilson et al. carried out a genome-wide analysis of the binding sites of ten key regulators of blood stem/progenitor cells and identified a combinatorial functional interaction between a heptad of transcription factors, including ERG (Table 1; [115]); the study also reported a direct physical interaction between ERG and Runt-Related transcription factor 1 (RUNX1) [115]. Dryden et al. identified a novel nuclear factor (NF)-κB/ETS consensus site involved in ERG-dependent repression of pro-inflammatory genes [24]. The authors showed that ERG blocks NF-κB p65 binding to the promoters of intercellular adhesion molecule (ICAM)-1, interleukin (IL)-8 and cellular inhibitor of apoptosis (cIAP)-2 in resting human umbilical vein endothelial cells (HUVEC); inhibition of ERG expression resulted in p65 binding to DNA and induction of NF-κB target gene expression.

A similar repression mechanism of interference was observed in prostate cancer cells, where Yu et al. found that ERG disrupts androgen receptor (AR) signalling by binding to and repressing AR downstream targets at gene-specific loci [119]. Co-immunoprecipitation assays demonstrated a physical interaction between the AR and ERG proteins in vCaP cells as well as prostate cancer tissues [119]. ERG also inhibits nuclear oestrogen receptor (ER)-α-dependent transcription; conversely, the transcriptional activity of ERG has been shown to be repressed by ERα, demonstrating a mutual repressive functional interaction between the two proteins [106]. In adult human endothelial cells, direct interaction and functional antagonism between ERG and ETS-2 has been reported, in which ERG interaction with ETS-2 inhibits the ability of ETS-2 to transactivate the matrix metalloprotease 3 (MMP3) promoter [14].

Recent studies have shown ERG's association with proteins that mediate its post-translation regulation (see also Section 7). Selvaraj et al. showed a high affinity interaction between ERG and ERK2 using microscale thermophoresis [87]. Wang et al. demonstrated that ubiquitin-specific peptidase 9, X-linked (USP9X), a deubiquitinase enzyme, binds ERG in VCaP prostate cancer cells expressing TMPRSS2-ERG and deubiquitinates ERG in vitro [111]. Furthermore, co-immunoprecipitation assays showed that endogenous ERG associates with speckle-type POZ protein (SPOP) ubiquitin ligase in LNCaP prostate cancer cells [2], [30] (Table 1; see also Section 7).

## 6 ERG expression and localization

In the developing mouse embryo, ERG is expressed from embryonic day (E)8.5 in mesodermal tissues, such as the endothelium, myocardium, pre-cartilage and haematopoietic tissues, but not in the epithelium or lymphocytes [65], [84], [104], [105]. ERG expression progressively decreases in the developing zebrafish vasculature; however in the mouse and human ERG remains highly expressed in EC of most adult tissues [6], [26], [36], [105], [120]. Genomic studies on EC from multiple origins have shown that ERG is the most highly expressed ETS factor in differentiated quiescent EC, with no major differences in levels between large arterial, venous and microvascular endothelium [9], [39].

Comprehensive characterization of ERG subcellular localization has shown that ERG is localized in the nucleus of endothelial cells [12]; indeed, many studies use ERG as nuclear marker for EC in mouse retinal vasculature [12], [28], [50]. Most studies have been carried out using anti-ERG antibodies which recognize epitopes within the C-terminus of the protein. The recently described N-terminal mouse monoclonal anti-ERG antibody (clone 9FY; [29]) can also detect ERG8, the isoform which lacks the nuclear localization sequence and which, in over-expression studies, has been shown to be localized in the cytoplasm (see Section 3; [38], [81]). Future studies using this and other tools will be able to investigate expression and subcellular localization of ERG8 in the endothelium.

## 7 Regulation of ERG expression and activity

The activity of many ETS factors is regulated by signal transduction cascades, which alter their sub-cellular localization, DNA binding activity, and/or transcriptional activity through post-translational modification. Little is known about the post-translational modifications of ERG in endothelial cells. In myeloblast cells, ERG is phosphorylated on a serine residue by an activator of the protein kinase C pathway [68], whereas in VCaP cells ERG is phosphorylated on serine residues at positions 81 and 215 (S81, S215), by both IκB and Akt kinases [93]. Recently, a study using arterial EC has indicated that ERG transcriptional activity can be regulated by VEGF/Mitogen-activated protein kinase (MAPK)-dependent signalling. Wythe et al. demonstrated that VEGF-mediated MAPK signalling drives expression of the Notch signalling pathway genes Dll4 and Notch4 by promoting ERG binding to their gene regulatory regions [116]. The differential ERG occupancy was not mediated by changes in total ERG levels or subcellular localization, and was inhibited by a MAPK inhibitor, suggesting that VEGF/MAPK signalling enhances the DNA binding activity of ERG in this context. Interestingly, several ETS family members are phosphorylated by MAPKs ([37]; [75], [69]) and these modifications are known to affect their interaction with other transcription factors as well as their binding to DNA [40]. Indeed, recent data from Selvaraj et al. using an in vitro cell-free screening assay revealed that ERG is predominantly phosphorylated at S215 by ERK2 kinase and that ERG phosphorylation was necessary for an overexpressing ERG retrovirus to drive migration of prostate epithelial cells [87]. These authors further demonstrated that ERK2-dependent phosphorylation increased ERG-dependent binding and transactivation of genes involved in epithelial cell migration. We have found that in quiescent, confluent HUVEC, ERG is also phosphorylated at serine residues, including S215 (S. Martin Almedina & A.M. Randi, unpublished data). The functional significance of ERG phosphorylation in EC is presently unknown.

Two recent studies have suggested that dysregulation of the SPOP ubiquitin ligase complex in ERG-overexpressing prostate cancer cells reduces ERG ubiquitination, and that stabilized ERG was responsible for the enhanced migration and invasion activities of cells carrying SPOP mutations [2], [30]. Whether this ubiquitin ligase system functions to regulate physiological ERG levels in endothelial cells is unknown. A role for ERG ubiquitination in prostate cancer cells was also demonstrated by Wang et al. who showed that the enzyme USP9X, which is highly expressed in ERG-positive prostate tumours, mediates ERG deubiquitination and thus its stabilization [111].

## 8 ERG-dependent gene targets and pathways in the endothelium

ERG regulates the expression of multiple EC genes with roles in key cellular functions such as survival, junction stability and cell migration; acting as a key regulator of endothelial homeostasis. A summary of ERG target genes and their role in endothelial cell function and homeostasis is shown in Table 2.

### 8.1 VEGF, Notch and arterial differentiation

Wythe et al. described a role for ERG in arterial specification, by demonstrating that ERG mediates VEGF-dependent expression of arterial Dll4, the earliest Notch ligand gene expressed in arterial precursor cells, during vascular development [116]. The Notch receptor Notch4 was also regulated by this VEGF/MAPK/ERG pathway. The authors reported increased ERG expression in arterial-derived EC in vitro; however, this is not in line with multiple studies on ERG mRNA and protein expression, in adult human and mouse tissue, as well as the embryonic and retinal mouse vasculature, showing that ERG is strongly expressed in all EC, with no detectable difference between arteries and veins [9], [12], [39], [52].

### 8.2 VE-cadherin, claudin-5, ICAM-2: cell permeability and junction integrity

ERG plays a key role in maintaining junction integrity through its transcriptional regulation of multiple junction molecules. ERG binds and transactivates the promoters of the endothelial junctional adhesion molecules VE-cadherin [10], claudin-5 [122] and ICAM-2 [61]. Inhibition of ERG expression in HUVEC results in a marked decrease in EC barrier function, which was partially rescued by adenoviral overexpression of claudin-5 [122]. Interestingly, over-expression of ERG could reduce permeability of VEGF-induced neovessels in vivo [12]. ERG is required for EC survival, partly via a pathway involving VE-cadherin and endothelial junction integrity [10]. In vivo, endothelial-specific deletion of ERG also results in reduced VE-cadherin expression in the postnatal retina [12].

### 8.3 Wnt/β-catenin signalling and vessel stability

Canonical Wnt signalling promotes EC survival, junction stabilization, proliferation and pericyte recruitment and is essential for vessel stability ([17]; reviewed in [20], [27]). The balance between VE-cadherin and Wnt-dependent signals controls β-catenin cellular localization and activity. Birdsey et al. showed that ERG controls the Wnt/β-catenin pathway by promoting β-catenin stability through transcriptional control of both VE-cadherin and the Wnt receptor Frizzled-4 [12]. The study also showed that ERG controls cell survival, proliferation, angiogenesis and vessel stability through β-catenin. Activation of Wnt signalling with lithium chloride, which stabilizes β-catenin levels, rescued sprouting and proliferation of ERG-deficient HUVEC in vitro and corrected vascular defects in endothelial-specific *Erg*-knockout embryos in vivo [12].

### 8.4 HDAC6 and RhoJ in migration and cytoskeletal dynamics

Transcriptome profiling of ERG-deficient EC identified ∼ 80 genes involved in cell migration as candidate ERG targets, including many regulators of the small GTPase Rho family [11]. Phalloidin-staining of ERG-deficient HUVEC revealed a marked alteration of both cell shape and actin stress fibre alignment [11], [122]. Additionally, in vitro scratch-wound migration assays and single cell imaging showed that inhibition of ERG decreases the speed and distance at which HUVEC migrate and results in a reduction of lamellipodia formation [11].

ERG has been shown to regulate the endothelial cytoskeleton through the activity of histone deacetylase-6 (HDAC6) [11] and the small GTPase RhoJ [121]. Inhibition of HDAC6 results in hyperacetylation of cortactin and α-tubulin (a marker of microtubule stabilization) leading to reduced EC migration and defects in in vitro and in vivo angiogenesis [45], [56]. Birdsey et al. showed that ERG drives constitutive HDAC6 expression in EC; following ERG inhibition the down-regulation of HDAC6 led to a dramatic increase in acetylated microtubules in HUVEC [11]. This observation was confirmed in vivo using ERG-siRNA in the Matrigel plug angiogenesis assay in mice; inhibition of ERG resulted in a reduction in endothelial HDAC6 expression, which coincided with increased tubulin acetylation compared to controls [11]. RhoJ is a GTPase belonging to the Cdc42 subfamily, which has been shown to be required for EC migration [46]. Yuan et al. identified RhoJ as a direct transcriptional target of ERG; using in vitro and in vivo tube formation assays, they also demonstrated a role for ERG and RhoJ during neovessel lumen formation [121].

## 9 Roles of ERG in the vasculature

### 9.1 ERG controls endothelial differentiation and reprogramming

ERG drives the expression of genes that define the endothelial lineage, such as VE-cadherin [10], [33], vWF [62], [85], endoglin [76] and eNOS [53]. Early studies in *Xenopus* showed a role for ERG in endothelial differentiation, where ectopic expression of the *Xenopus* homolog of ERG drove ectopic endothelial differentiation in the ventral region of *Xenopus* embryos [6].

A further line of evidence for the key role ERG plays in endothelial differentiation comes from developmental studies of differentiation of embryoid bodies, which show that ERG is required for the differentiation of embryonic stem cells along the endothelial lineage [71]. Interestingly, a recent study has shown that constitutive expression of ERG and FLI-1 in combination with TGFβ pathway inhibition is sufficient to reprogramme non-vascular amniotic cells into stable vascular endothelial cells [31]. A recent study by Batta et al. demonstrated that both embryonic and adult somatic fibroblasts can be efficiently reprogrammed to haematopoietic progenitors by concomitant ectopic expression of ERG and other haematopoietic transcription factors (GATA2, LMO2, RUNX1c and SCL; [8]). Furthermore, Morita et al. demonstrated that ectopic expression of the ETS factor ETV2 induces expression of ERG in human fibroblasts and consequently ETV2-expressing fibroblasts convert into functional EC [66].

### 9.2 Regulation of vascular development by ERG

The role of ERG in vascular development has been demonstrated in multiple in vivo models. In the developing *Xenopus* embryo, ERG transcripts are detected in the vitelline veins, posterior cardinal veins, blood vessels of the head, along with strong ERG expression in the intersomitic blood vessels [6]. Over-expression of ERG in the *Xenopus* embryo resulted in developmental defects and ectopic endothelial differentiation. In zebrafish embryos, ERG antisense morpholino caused defective intersomitic vessel patterning and haemorrhage in the head [57]. However, combinatorial knockdown of ERG and other ETS factors, FLI-1 or ETV2, was required to cause severe vascular defects, suggesting a synergistic role for these ETS factors during zebrafish vascular development [26], [57].

**Fig. 2:**
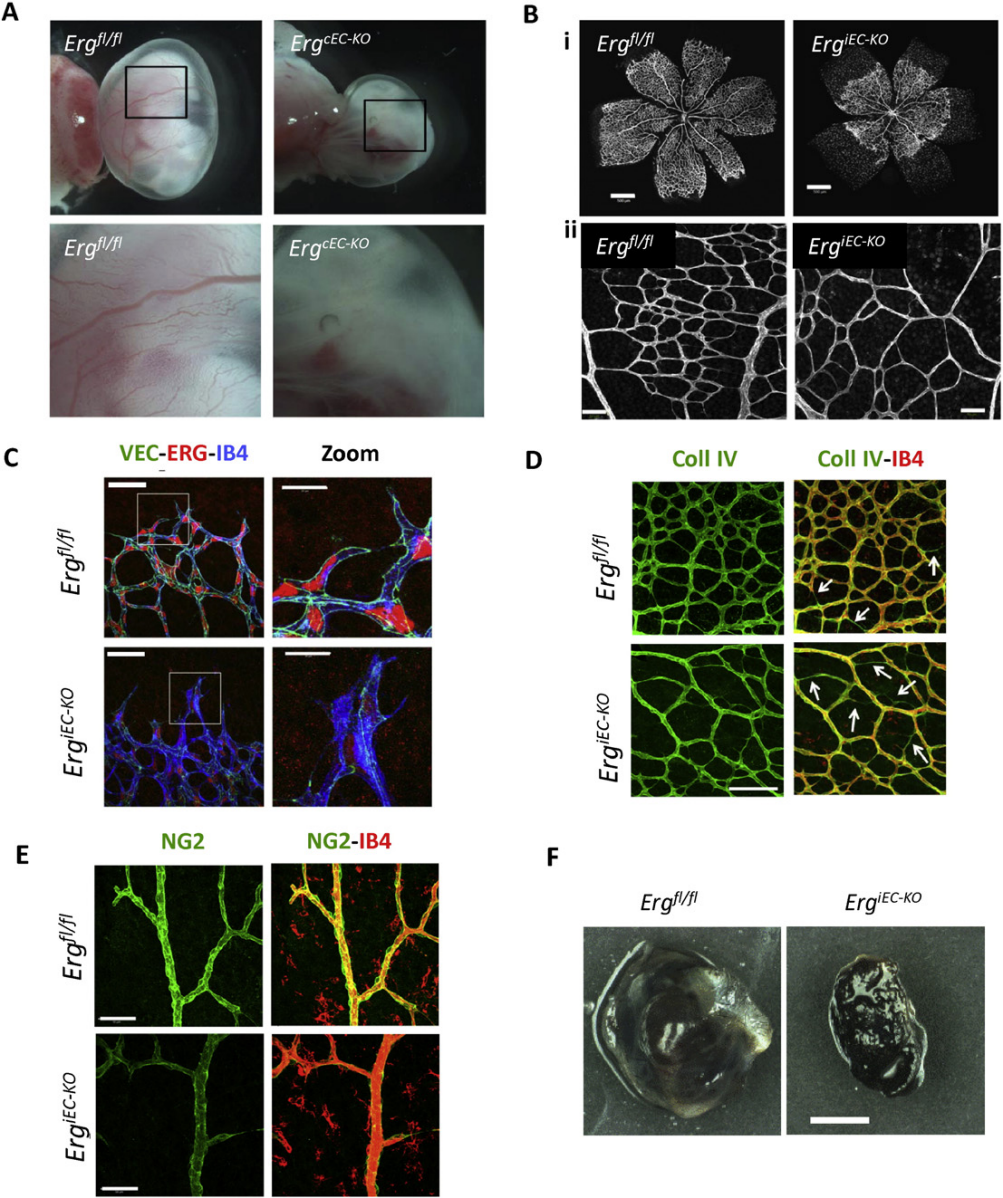
In vivo evidence of the role of ERG in the vasculature. (A) Light microscopy of the yolk sac surrounding E10.5 embryos reveals a decrease in yolk sac vascularization in *Erg*^*cEC-KO*^ embryos, compared to *Erg*^*fl*/*fl*^ controls. (B) Isolectin B4 staining of postnatal day 6 retinas show a reduction in (i) the overall extent of the vascular plexus and (ii) the number of vascular branches in *Erg*^*iEC-KO*^ mice compared to controls. (C) Staining for VE-cadherin (green), ERG (red) and isolectin B4 (IB4, blue) in *Erg*^*iEC-KO*^ and *Erg*^*fl*/*fl*^ P6 retinas. Scale bar, 50 μm; zoom, 20 μm. A marked reduction in VE-cadherin expression and junctional localization is observed in the retinal vasculature of *Erg*^*iEC-KO*^ mice. (D) Collagen IV (Coll IV; green) and isolectin B4 (IB4, red) staining of *Erg*^*iEC-KO*^ and *Erg*^*fl*/*fl*^ P6 retinal vessels. The capillary plexus in *Erg*^*iEC-KO*^ retinas show a greater number of empty collagen IV sleeves (arrows), indicating increased vessel regression. (E) NG2-positive pericytes (green) associated with isolectin B4 labeled retinal vessels (red) from *Erg*^*iEC-KO*^ and *Erg*^*fl*/*fl*^ mice shows that pericyte recruitment was significantly decreased along *Erg*^*iEC-KO*^ mouse vessels. (F) Representative images of B16F0 tumours from adult *Erg*^*iEC-KO*^ and *Erg*^*fl*/*fl*^ mice. Scale bar, 2 mm. Tumour size was significantly reduced in adult *Erg*^*iEC-KO*^ mice (images reproduced from Ref. [12], under the Creative Commons BY license; http://creativecommons.org/licenses/by/3.0/).

Two recent studies have used genetic lineage-specific ERG deletion in mice by crossing *Erg* floxed mice with *Tie2-Cre* mice [12], [34]. Constitutive homozygous deletion of endothelial *Erg* in the mouse embryo (*Erg*^*cEC-KO*^) caused embryonic lethality between E10.5 and E12.5, with severe disruption to the cardiovascular system, associated with defective vascular remodelling and haemorrhaging (Fig. 2A; [12], [34]). Importantly, Birdsey et al. showed that ERG controls vascular development in a Wnt/β-catenin-dependent manner, as in vivo LiCl treatment rescued the yolk sac vascular defects in the *Erg*^*cEC-KO*^ mice ([12], also see Section 8.3).

The vascular defects due to constitutive endothelial-specific deletion of ERG are in line with the study by Vijayaraj et al., where global deletion of a subset of ERG isoforms, shown to have predominantly endothelial expression, also resulted in cardiovascular defects and embryonic lethality at E11.5 [104]. The cardiac defects in these embryos were associated with a failure in endocardial–mesenchymal transition (EndMT) during cardiac valve morphogenesis, possibly linked to the ERG-dependent regulation of members of the Snail family of transcription factors [104].

Interestingly, a previous transgenic model where ERG's function was disrupted by a mutation in the DNA binding ETS domain (*Erg*^*Mld2*/*Mld2*^) caused embryonic lethality at a later stage (E13.5) [58] and did not appear to display early vascular defects, suggesting that ERG's functions in the vasculature are not exclusively mediated by its DNA binding activity. Instead, inhibiting ERG transactivation showed multiple defects in definitive haematopoiesis and a failure to sustain self-renewal of haematopoietic stem cells, pointing to an additional regulatory role for ERG during murine haematopoiesis ([58], [99], also see Section 10.1).

Surprisingly, a study by Lathen at al. [52] reported a distinctly different phenotype caused by *Cre*-mediated global deletion of ERG. In contrast with three separate studies which, using different genetic strategies, showed that deletion of endothelial ERG results in severe vascular defects and embryo lethality between E10.5 and E12.5 (see above, [12], [34], [104]), Lathen et al. reported that *Cre*-mediated global deletion of ERG caused delayed embryonic lethality, from E16.5 to 3 months of age. Vascular defects occurring after E14.5 were apparent in some ERG mutants, with oedema and subcutaneous haemorrhage [52]. Interestingly, mice with global deletion of ERG appear to develop pulmonary hypertension due to the onset of pulmonary veno-occlusive disease (PVOD). The discrepancies in the phenotypes between the global ERG-deficient mouse line and the multiple endothelial-specific lines reported are puzzling and could be due to technical variation; alternatively, global loss of ERG might result in compensation mechanisms that reduce the severity of vascular function during early development. More studies on global ERG deficiency will be required to clarify these discrepancies.

### 9.3 ERG is required for physiological and pathological angiogenesis

Studies using an inducible endothelial-specific ERG knockout mouse (*Erg*^*iEC-KO*^) have demonstrated that postnatal deletion of ERG results in defective retinal angiogenesis (Fig. 2B; [12]). ERG deficiency in retinal endothelial cells leads to reduced VE-cadherin expression (Fig. 2C), increased vessel regression (Fig. 2D) and reduced pericyte recruitment (Fig. 2E), in agreement with a role for ERG in the control of vascular stability during physiological angiogenesis [12].

Although aberrantly expressed ERG fusion proteins are associated with a number of different cancers (see Section 10.3), little information exists on the role of ERG in regulating tumour neovascularization. Recently, using a xenograft tumour model, Birdsey et al. demonstrated that deletion of endothelial ERG in the adult mouse significantly reduced the size of B16 melanoma tumours (Fig. 2F) and this was accompanied by a significant reduction in tumour blood vessel density and pericyte coverage of blood vessels [12].

### 9.4 ERG as a repressor of vascular inflammation

In line with its role in promoting vascular homeostasis, ERG expression is down-regulated by inflammatory stimuli such as tumour necrosis factor (TNF)-α, lipopolysaccharide (LPS) and interleukin-1β (IL-1β) [47], [61], [95], [120]. Moreover, ERG expression was lost from the endothelium overlaying the shoulder regions of human coronary plaques, known to be associated with inflammatory infiltrate and endothelial activation [95]. The modulation of ERG expression by pro-inflammatory stimuli suggests that its regulation may be critical during inflammatory processes. Indeed, several studies have described the role of ERG in repressing vascular inflammation. ERG has been shown to act as a gatekeeper to maintain the endothelium in an anti-inflammatory state, by repressing expression of pro-inflammatory molecules such as ICAM-1, vascular cell adhesion molecule (VCAM), plasminogen activator inhibitor (PAI)-1 and interleukin (IL)-8 [24], [95], [120]. ICAM-1 repression by ERG was due to inhibition of NF-κB p65 binding to the ICAM-1 promoter, suggesting a direct mechanism of interference [24]. Gene set enrichment analysis of ERG- and NF-κB-dependent genes identified by microarray analysis revealed that this mechanism is common to other pro-inflammatory genes, including IL-8 [24]. Functionally, ERG was able to inhibit in vitro leukocyte adhesion [95], [120] and transmigration (N. Dufton & A. Randi, unpublished data). In vivo, the functional relevance of ERG's anti-inflammatory role was demonstrated using a murine model of TNF-α-dependent acute inflammation, where over-expression of ERG in the mouse paw decreased TNF-α-induced paw swelling [95].

## 10 Physiological and pathological non-vascular roles of ERG

### 10.1 Haematopoiesis

Endogenously expressed ERG is found in megakaryocytes [78], chondrocytes [41] and premature T and B-lymphocytes [3]. ERG is transiently expressed during the early stages of T and B cell differentiation but is silenced permanently after T and B cell lineage commitment [3]. ERG is also required for definitive haematopoiesis, adult haematopoietic stem cell function, normal megakaryopoiesis and the maintenance of peripheral blood platelet numbers [58], [70], [99].

### 10.2 Bone and cartilage development

A role for ERG in limb skeletogenesis has been described. Dhordain et al. provided the initial evidence that ERG is expressed at sites of future synovial joint formation in chick embryo limbs [23]. Since then, studies have shown that ERG is selectively expressed in articular chondrocytes during mouse and chicken bone development [41], [42], [43]. ERG is induced by the bone morphogenetic protein Gdf5 and is highly expressed in regions of the articular cartilage that express lubricin [41]. Interestingly, overexpression of ERG in developing chick limbs effectively blocks chondrocyte maturation and endochondral ossification by maintaining the entire limb chondrocyte population in an immature state [41]. Vijayaraj et al. have shown that a subset of ERG isoforms, which share a common translational start site encoded by exon 3, are enriched in chondrocytes [104].

### 10.3 Cancer

Accumulating evidence points to ERG as a lineage-determining transcription factor; therefore its ectopic expression can be detrimental. Indeed, ERG ectopic expression has been linked to the pathogenesis of multiple cancers.

#### 10.3.1 Ewing sarcoma and leukaemias

Chromosomal translocations that result in the expression of oncogenic ERG fusion proteins have been identified in multiple malignancies. In Ewing sarcoma and acute myeloid leukaemia, chromosomal translocations result in fusion of ERG with the RNA binding proteins EWS and FUS, respectively, producing chimeric proteins [74], [90], [91], [94]. The EWS and FUS genes are closely related and contain conserved domains [21]. The most common fusions in Ewing sarcoma actually occur between EWS and FLI-1 (85%), while the EWS:ERG fusion has a 5–10% occurrence rate. In Ewing sarcoma, ERG fusions result in replacement of the C-terminus of EWS by the DNA-binding domain of ERG resulting in loss of endogenous ERG promoter activity, causing dysregulation of ERG and its target genes [7]. High expression of ERG is a poor prognostic indicator in both acute myeloid leukaemia and acute lymphoid leukaemia [5], [60] and increased ERG mRNA expression has been observed in acute myeloid leukaemia patients with complex karyotypes and abnormal chromosome 21 [4]. ERG maps to the Down's syndrome critical region of chromosome 21, where an increase from diploid to triploid gene dosage has been implicated in Down's syndrome-associated megakaryocytic leukaemia [69], [78].

#### 10.3.2 Prostate cancer

More than 50% of all prostate cancers harbour a chromosomal translocation that results in the fusion of the androgen receptor-regulated gene promoter of transmembrane protease serine (TMPRSS)-2 and ERG [102]. This translocation leads to aberrant overexpression of nearly the entire ERG protein, including the DNA-binding domain, in the prostate epithelium. In addition, over-expressed TMPRSS2:ERG fusion protein is able to induce expression of native ERG through activation of one of the three native ERG promoters [59]. How the fusion products regulate prostate cancer remains unclear, although it has been observed that an increased incidence of the TMPRSS2:ERG fusion protein in prostate epithelial cells correlates with increased cell invasiveness, poor prognosis and higher rates of malignancy [101]. In combination with deletion of the Phosphatase and Tensin Homolog (PTEN) or up-regulation of the oncogenic serine/threonine protein kinase Akt, ERG overexpression induces progression to prostate cancer [96]. The role of ERG-fusion proteins in prostate cancer has been reviewed in detail elsewhere [1].

##### 10.3.2.1 microRNAs and prostate cancer

Several studies have examined correlation between ERG and micro-RNAs (miRNAs) in prostate cancer. Hart et al. showed that miR-145, which is down-regulated in prostate cancer, inhibits ERG expression by directly targeting its 3′UTR [35]. Thus, loss of miR-145 may provide a TMPRSS2-ERG gene fusion-independent means to up-regulate ERG expression in prostate cancer. Analysis of prostate cancer samples also showed that miR-221 is down-regulated in patients with TMPRSS2-ERG gene fusion-positive tumours compared to ERG fusion negative samples [32]. By integrating ERG ChIP-seq data with miRNA profiling data in ERG-fusion positive prostate cancer cells, Kim et al. identified miR-200c as a putative downstream miRNA regulated by ERG. The authors also demonstrated that miR-200c is a direct target of ERG and is repressed in ERG fusion-positive prostate cancer. In addition, they showed that miR-200c loss mediates ERG-induced epithelial-to-mesenchymal transition and cell motility [48].

#### 10.3.3 Vascular malignancies

ERG has been shown to be both a sensitive and specific marker for endothelial cells in vascular malignancies, including angiosarcoma, haemangioma, lymphangioma, Kaposi sarcoma, and haemangioendothelioma [64]. Whether ERG plays an oncogenic role in vascular tumours is unknown.

## 11 Concluding remarks

The study of the role of ERG in vascular development and angiogenesis has had an upsurge in recent years. It is now clear that ERG is essential for differentiation and maintenance of the endothelial lineage, and therefore for the development and maintenance of healthy vasculature. This is in striking contrast with its role in promoting oncogenesis when ectopically expressed. Although substantial progress in understanding the function of ERG has been made, much remains to be discovered. Upcoming areas of study will include the identification of binding partners that regulate ERG activity, the regulation of ERG function by post-translational modifications and by upstream signals. Understanding the homeostatic function of ERG in endothelial cells will provide insight into novel approaches to promote vascular health, as well as possible therapeutic options to selectively target the oncogenic function of ERG in cancer.

## Funding

GMB and AMR are supported by the British Heart Foundation (RG/11/17/29256).

## Figures and Tables

**Table 1:** ERG binding and functional partners. ERG is able to associate with a wide variety of binding partners which will have functional implications for regulating cellular responses. In most cases, interactions involving nuclear proteins modulate transcriptional activity of either ERG or the associated protein. ERG also has a number of functional interaction partners, where no direct binding data has been provided.

Binding partner		Methods	References
ETS factors	ERG	GST pull down, co-immunoprecipitation	[16]
	ETS-2		
	FLI-1		
	ER81		
	PU-1		
Other transcription factors	AP-1	GST pull down, co-immunoprecipitation, fluorescence resonance energy transfer microscopy (FRET)	[15], [16], [103]
	KLF2	Co-immunoprecipitation	[63]
	RUNX1	Co-immunoprecipitation	[115]
	Xvent2	Yeast two-hybrid screen, GST pull down	[22]
	Xvent2B		
Nuclear receptors	AR	GST pull down, co-immunoprecipitation	[119]
DNA damage repair proteins/Co-factors	DNA-PKcs	Mass spectrometry, co-immunoprecipitation	[13]
	Ku70		
	Ku80		
	PARP1		
Histone methyltransferase	ESET	Yeast two-hybrid screen, GST pull down, co-immunoprecipitation	[117]
Ubiquitin ligases	UBC9		
	SPOP	Co-immunoprecipitation	[2], [30]
Deubiquitinase enzyme	USP9X	GST pull down, co-immunoprecipitation, mass spectrometry	[111]
Serine threonine kinase	ERK-2	Microscale thermophoresis	[87]
Splicing factor	RNP C	Yeast two-hybrid screen, GST pull down	[22]


Functional partner		Methods	References
Transcription factors	P65	Chromatin immunoprecipitation, electrophoretic mobility shift assay, transactivation reporter assay	[24]
	SCL	Chromatin immunoprecipitation with high-throughput DNA sequencing	[115]
	LYL1		
	LMO2		
	GATA2		
Transcriptional co-activators	P300	Transactivation reporter assay	[44]
Nuclear receptors	ERα	Transactivation reporter assay	[106]

**Table 2:** Endothelial ERG target genes.

Genes activated by ERG			
Functional categories	Gene	Name	References
Endothelial homeostasis			
	APLNR	Apelin receptor	[52]
	NOS3	Endothelial nitric oxide synthase (eNOS)	[53]
	NOTCH4	Notch 4	[116]
	DLL4	Delta-like ligand 4	[116]
	ENG	Endoglin	[76]
	HMOX1	Haem oxygenase 1	[22]
	SNAI1	Snail family zinc finger 1	[104]
	SNAI2	Snail family zinc finger 2	[104]

Endothelial cell–cell junctions			
	CDH5	Vascular endothelial (VE)-cadherin	[10], [33]
	CLDN5	Claudin-5	[122]
	ICAM2	Intercellular adhesion molecule 2	[61]

Angiogenesis			
	FLK1	Vascular endothelial growth factor receptor 2 (VEGFR2)	[63]
	FLT1	Vascular endothelial growth factor receptor 1 (VEGFR1)	[107]
	FZD4	Frizzled class receptor 4	[12]
	EGFL7	EGF-Like protein 7	[54]

Cytoskeleton dynamics; cell migration			
	HDAC6	Histone deacetylase 6	[11]
	RHOA	Ras homolog family member A	[62]
	RHOJ	Ras homolog family member J	[121]

Extracellular matrix			
	MMP1	Collagenase 1	[14]
	SPARC	Secreted protein acidic and cysteine rich	[62]
	TSP1	Thrombospondin	[62]
Haemostasis/thrombosis			
	VWF	Von Willebrand factor	[62], [85]


Genes repressed by ERG			
Functional categories	Gene	Name	References
Apoptosis			
	BIRC3	Cellular inhibitor of apoptosis 2 (cIAP2)	[24]

Vascular inflammation			
	CD44	CD44	[120]
	CXCL8	Interleukin-8 (IL8)	[120]
	ICAM1	Intercellular adhesion molecule 1	[24], [95]
	MMP3	Stromelysin 1	[14]
	SERPINE1	Plasminogen activator inhibitor 1 (PAI1)	[120]
	VCAM1	Vascular cell adhesion molecule 1	[95]

Extracellular matrix degradation			
	PLAU	Plasminogen activator, urokinase	[120]
